# Antiviral Activity of a Cyclic Pro-Pro-*β^3^*-HoPhe-Phe Tetrapeptide against HSV-1 and HAdV-5

**DOI:** 10.3390/molecules27113552

**Published:** 2022-05-31

**Authors:** Ewa Zaczyńska, Krzysztof Kaczmarek, Janusz Zabrocki, Jolanta Artym, Michał Zimecki

**Affiliations:** 1 Department of Experimental Therapy, Hirszfeld Institute of Immunology and Experimental Therapy, Polish Academy of Sciences, Laboratory of Immunobiology, R. Weigla Str. 12, 53-114 Wrocław, Poland; ewa.zaczynska@hirszfeld.pl (E.Z.); jolanta.artym@hirszfeld.pl (J.A.); 2 Institute of Organic Chemistry, Lodz University of Technology, S. Żeromskiego Str. 116, 90-924 Łódź, Poland; krzysztof.kaczmarek@p.lodz.pl (K.K.); janusz.zabrocki@p.lodz.pl (J.Z.)

**Keywords:** cyclic tetrapeptide cyclo(Pro-Pro-*β^3^*-HoPhe-Phe), A-549, HAdV-5, HSV-1, antiviral activity

## Abstract

The core of Cyclolinopeptide A (CLA, cyclo(LIILVPPFF)), responsible for its high immunosuppressive activity, contains a Pro-Pro-Phe-Phe sequence. A newly synthesized cyclic tetrapeptide, cyclo(Pro-Pro-*β^3^*-HoPhe-Phe) (denoted as 4B8M) bearing the active sequence of CLA, was recently shown to exhibit a wide array of anti-inflammatory properties in mouse models. In this investigation, we demonstrate that the peptide significantly inhibits the replication of human adenovirus C serotype 5 (HAdV-5) and *Herpes simplex* virus type-1 (HSV-1) in epithelial lung cell line A-549, applying Cidofovir and Acyclovir as reference drugs. Based on a previously established mechanism of its action, we propose that the peptide may inhibit virus replication by the induction of PGE2 acting via EP2/EP4 receptors in epithelial cells. In summary, we reveal a new, antiviral property of this anti-inflammatory peptide.

## 1. Introduction

The search for new immunosuppressive and anti-infectious therapeutics, both synthetic and derived from natural sources, has intensified. Among compounds of potential therapeutic utility are cyclic peptides [1,2,3], displaying a variety of immunosuppressive, anti-inflammatory and antimicrobial properties, which are less toxic than conventional immune suppressors. Cyclolinopeptide (CLA), isolated from linen seeds, and its derivatives, is also attractive [4] as a potential therapeutic. Recently, the studies on CLA derivatives resulted in the synthesis of a cyclic tetrapeptide, bearing a Pro-Pro-*β^3^*-HoPhe-Phe sequence, referred to below as the 4B8M peptide. 4B8M exhibited anti-inflammatory actions in several in vivo mouse models, such as antigen-specific skin inflammatory reactions and responses to nonspecific irritants, colon inflammation induced by dextran sulfate and allergic pleurisy induced by ovalbumin [5]. The concept of constructing such a peptide derived from our earlier studies on CLA analogs indicated that the inclusion of tetrapeptidic (Pro-Pro-Phe-Phe) or tripeptidic (Pro-Phe-Phe) fragments in longer linear peptide chains seems to have crucial significance for immunosuppressive activity [6,7]. The mechanism of action of the 4B8M peptide was associated with its ability to induce cyclooxygenase 2 (COX-2) production in a keratinocyte cell line and differential effects on lipopolysaccharide (LPS)-induced expression of prostaglandin E2 (PGE2) receptors: EP1 and EP3 [5]. Of interest, no antimicrobial properties of CLA analogs have been reported to date. Nevertheless, among cyclic di-, tetra- and pentapeptides, such actions have been found [1,8,9,10]. Pentapeptides may act as ligands in the influenza virus hemagglutinin structure cavity, thus preventing its fusion with the endosome membrane [10]. Cyclic peptides may also serve as antagonists of C-X-C chemokine-receptor type 4 (CXCR4), serving as a co-receptor for viral entry [11,12]. CXCR4 is a receptor for stromal cell-derived factor 1 and is a key molecule in initiation of signaling pathways responsible for major physiological and pathogenic processes [13]. It is also a potential therapeutic target in hematologic tumors [14].

Based on the above reports, we hypothesized that 4B8M peptide may also exhibit antiviral properties, and we decided to investigate its effect on the replication of two types of viruses in epithelial lung cell line A-549. Human adenoviruses (HAdVs) have been implicated as infectious agents responsible for numerous diseases, including respiratory tract infections, as well as ocular and gastrointestinal tract disorders [15]. Adenoviruses usually cause mild, self-limiting respiratory illnesses, primarily in children, due to normal host responses that include a natural innate immune response involving the induction of cytokines and activation of effectual leukocytes [16]. However, potentially fatal disseminated diseases in highly immunocompromised patients have also been reported, particularly in pediatric bone marrow transplant recipients. *Herpes simplex* virus (HSV), on the other hand, is an enveloped pathogen from the *Herpesviridae* family, which causes a broad range of infections. HSV type 1 (HSV-1) is involved in a variety of lesions in mucous membranes, and especially manifests in immunodeficient patients [17]. This type of infection is recurrent and patients who use licensed drugs often experience severe side effects and prevalent resistance [18].

The aim of this study is to evaluate the potential antiviral properties of 4B8M using epithelial lung cell line A-549 infected with human adenovirus C serotype 5 (HAdV-5) and HSV-1. To compare the potential antiviral property of 4B8M, we applied respective, clinically approved reference drugs, such as Cidofovir and Acyclovir. Cidofovir [(*S*)-HPMPC; (*S*)-1-(3-hydroxy-2-phosphonylmethoxypropyl)cytosine)] is an acyclic nucleoside phosphonate that shows antiviral activity against DNA viruses [19]. It inhibits HAdV replication, independent of the serotype, by acting as a monophosphate form of nucleotide. Acyclovir and its sodium salt, in turn, were the first effective antiviral agents against *Herpes simplex* viruses (HSV-1 and -2). Acyclovir is made from an acyclic nucleoside analogue, which incorporates into viral DNA inside an infected mammalian cell, where it inhibits viral replication [20]. The results presented in this work reveal that 4B8M exhibits potent antiviral properties in the above described models.

## 2. Results

### 2.1. Toxicity of the 4B8M Peptide

**Figure 1:**
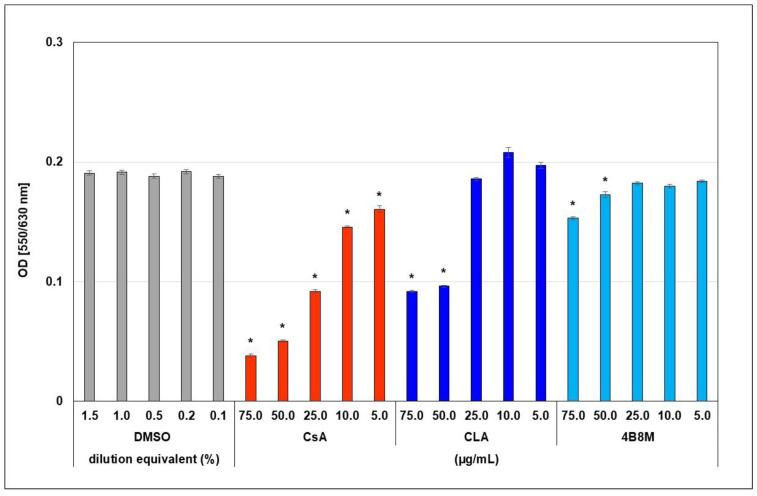
Toxicity of 4B8M against mouse splenocytes in comparison to related compounds. Splenocytes were cultured for 24 h with the compounds at indicated concentrations. Control cultures contained corresponding dilutions of the solvent (DMSO). Cell survival was determined by the MTT colorimetric method. The results of the cytotoxic tests are presented as mean values of optical density (OD) at 550/630 nm ± standard error (SE). * *p* < 0.05 as compared with appropriate DMSO controls.

Low cell toxicity is a desirable feature of potential therapeutic drugs. Therefore, we evaluated the cytotoxic effect 4B8M, together with reference compounds, in the 24 h cultures of mouse splenocytes (Figure 1). For the experiment, we used CLA—a parent peptide for 4B8M and cyclosporine (CsA), since CLA shares the mechanism of action with CsA [21]. In addition, CsA also exhibits antiviral activity [22]. The compounds were tested at the 5–75 µg/mL concentration range. Cell viability was determined using the MTT colorimetric method. The results clearly reveal the strong, dose-dependent toxicity of CsA, already at 25 µg/mL (51 % cell death). The toxicity of CLA was also substantial and evident at 50 µg/mL (50% cell death). In contrast, 4B8M demonstrated only a very low toxicity (10%) at 50 µg/mL and only 22% inhibition of cell survival at 75 µg/mL.

**Figure 2:**
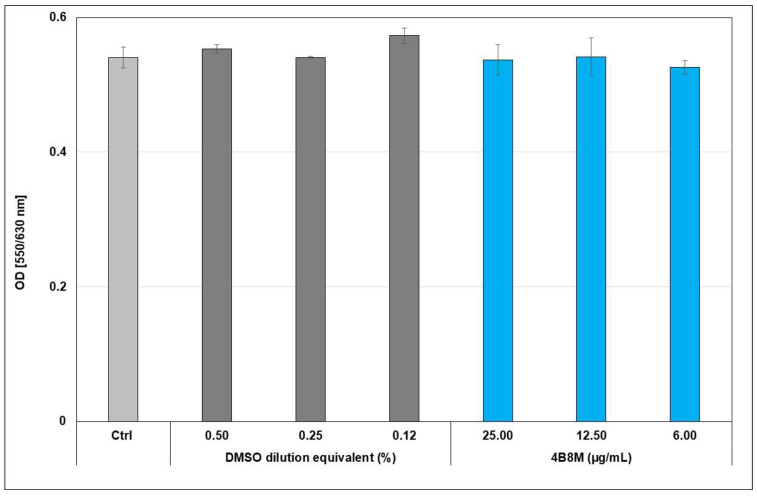
Cell toxicity of 4B8M against reference A-549 cell line. The test was performed in a monolayer culture of cell line A-549 that was incubated at a density of 5×10^4^/well for 72 h. 4B8M was tested at a 6–25 μg/mL concentration range. Control cultures contained corresponding dilutions of the solvent (DMSO) or medium only—Ctrl. Cell viability was determined by the MTT colorimetric assay. The results are presented as OD mean values ± SE from quadruplicate determinations.

We also evaluated whether the peptide lowers the viability of the target A-549 cell line. The cell line was incubated for 72 h at a 6–25 µg/mL concentration range of the peptide, and cell viability was assessed by MTT colorimetric assay (Figure 2). No change in cell viability was observed up to a 25 µg/mL concentration. Our unpublished studies also showed that the 4B8M peptide was not toxic with regard to human peripheral blood mononuclear cells, even at a concentration of 100 µg/mL. 

**Figure 3:**
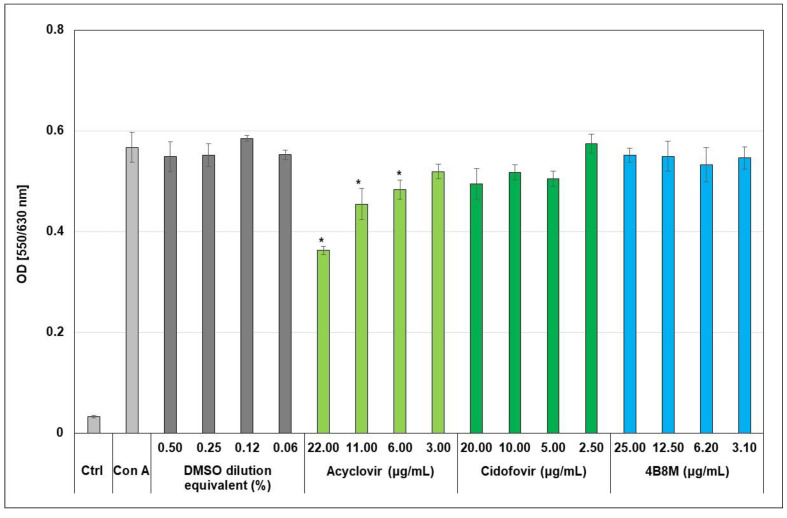
Effects of 4B8M and the reference compounds on concanavalin A (ConA)-induced splenocyte proliferation. 4B8M and the reference drugs were added at the indicated concentrations. Control cultures contained the solvent (DMSO) at dilutions equivalent to those present in respective doses of the studied compounds or medium only—Ctrl. All cultures contained ConA (2.5 µg/mL), except Ctrl. Following a 3-day incubation period, cell proliferation was determined using the MTT colorimetric assay. The results are presented as OD mean values ± SE from quadruplicate determinations. * *p* < 0.05 as compared with appropriate controls.

Considering the mechanism of action of classical antiviral drugs [20], they should also affect the replication of nucleic acids and, consequently, the cell division of host cells. Therefore, we evaluated the effects of the 4B8M peptide and reference drugs on mitogen-induced proliferation of mouse splenocytes (Figure 3). The results show that Acyclovir, up to a concentration of 6 µg/mL, inhibits splenocyte proliferation in a statistically significant manner (34, 22 and 12.5 % cell death for concentrations of 22, 11 and 6 µg/mL, respectively). Cell proliferation by Cidofovir was only slightly inhibited at this concentration range. 4B8M, on the other hand, did not affect cell proliferation in this model.

### 2.2. The Virus-Inhibiting Properties of the 4B8M Peptide against Human HAdV-5

**Figure 4:**
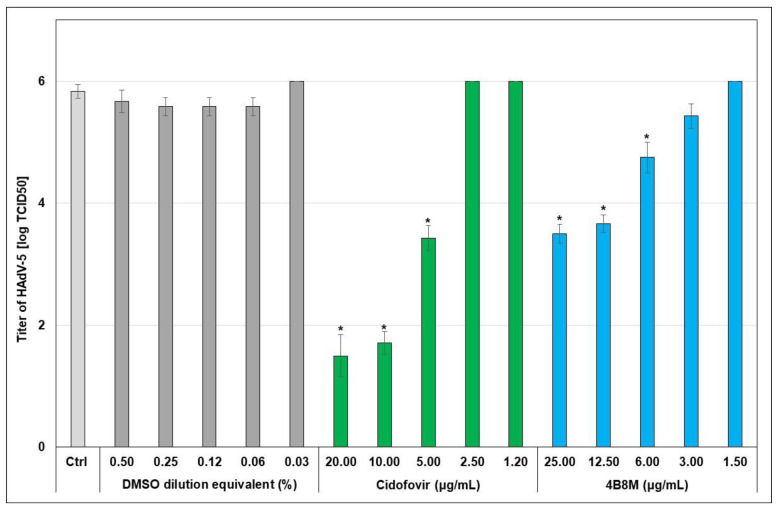
The effect of 4B8M on HAdV-5 replication in A-549 cells. A-549 cells were infected with HAdV-5 of MOI = 1 and, after 1 h of virus absorption at 37 °C, the virus inoculums were removed and the infected cells were incubated for 48 h with the peptide (25; 12.5; 6.2 and 3.1 μg/mL) or DMSO as the solvent control for 4B8M or in medium only (Ctrl). Cidofovir (20.0; 10.0; 5.0; 2.5 and 1.2 μg/mL) was used as a reference drug. The viral titer was expressed with reference to the TCID50 value, which is based on the cytopathic effect caused by this virus in approximately 50% of infected cells. The results, obtained from 4 independent experiments (mean ± SE), are presented as the logTDIC50 values in relation to appropriate DMSO control. * *p* < 0.05 as compared with appropriate controls.

**Figure 5:**
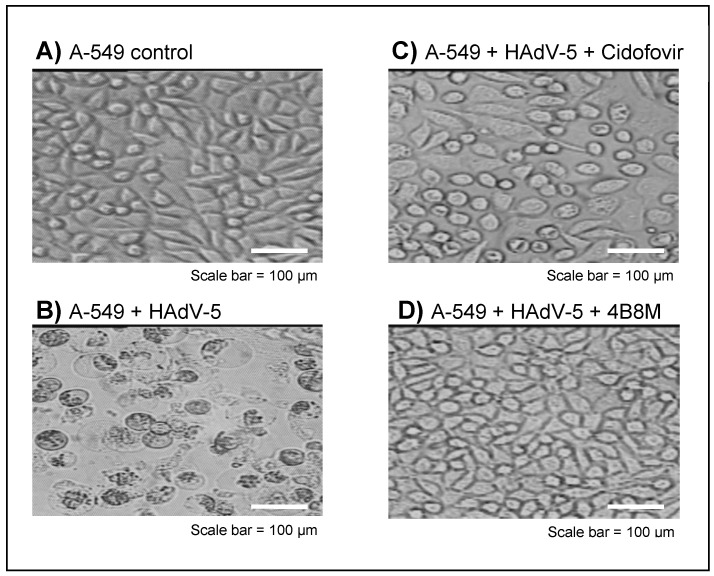
Antiviral activity of 4B8M against HAdV-5 and its morphological changes in the A-549 cells examined with phase contrast microscopy. The photographs show 48 h cultures of A-549 cells infected with HAdV-5 virus (magnification 300×); (**A**) photograph shows a normal appearance of the control culture of A-549 cells in the culture medium, (**B**–**D**) photographs show cells pretreated with the virus: (**B**) HAdV-5 negative control; after 48 h infection with HAdV-5, the A-549 cells showed some morphological changes, including loss of monolayer integrity, lyses, granulation, vacuolization in the cytoplasm, (**C**) A-549 cells infected with HAdV-5 in the presence of Cidofovir (20 µg/mL), (**D**) A-549 cells after 48 h co-incubation with HAdV-5 and 4B8M (25 µg/mL).

Subsequently, the 4B8M peptide’s potential ability to inhibit viral replication was investigated in the model of A-549 cells infected with the HAdV-5 virus, using Cidofovir as a reference drug. The results, with a description of the experiments, are presented in Figure 4. To determine whether the 4B8M peptide has antiviral activity against HAdV-5, we infected A-549 cells with HAdV-5 (MOI = 1) and then treated the cells with different concentrations of the compound for 48 h. As shown in Figure 4, 4B8M inhibited viral titers in a dose-dependent manner. The treatment of A-549 cells infected with HAdV-5 with 4B8M resulted in a significant reduction in virus titers reaching a 2.2-log and 1.9-log decrease for 4B8M at concentrations of 25.0 µg/mL and 12.5 μg/mL, respectively, and 0.8-log for 6 μg/mL of 4B8M the—effects were statistically significant. The efficacy of Cidofovir was stronger and attained its maximal value at 20.0 µg/mL—viral titers were reduced to 1.5-log (Figure 4). Of note, when Cidofovir lost its activity at 2.5 µg/mL, 4B8M at 3 µg/mL still exhibited some antiviral action. The above presented results are supplemented by a visualization of the effects of the peptide and the reference drug on the morphology of infected cells (Figure 5). The photographs show an improvement of cell disintegration caused by the virus, following incubation with the investigated compounds at the indicated concentrations.

### 2.3. The Virus-Inhibiting Properties of 4B8M Peptide against Human HSV-1

**Figure 6:**
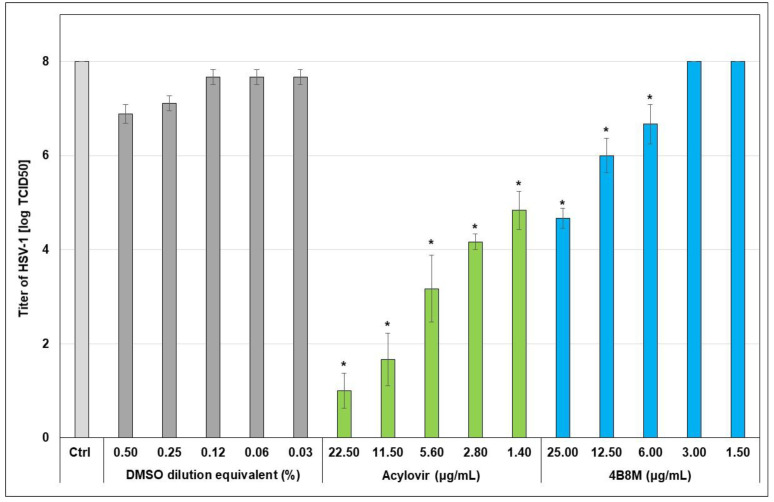
The effect of 4B8M on HSV-1 virus replication in A-549 cells. A-549 cells were infected with HSV-1 of MOI = 4 and, after 1 h of virus absorption at 37 °C, the virus inoculum was removed and the infected cells were incubated for 48 h with 4B8M (25.0; 12.5; 6.0; 3.0; 1.5 μg/mL), DMSO as the solvent control for 4B8M or medium only (Ctrl). Acyclovir (22.5; 11.2; 5.6; 2.8 and 1.4 μg/mL) was used as a reference drug. The viral titer was expressed with reference to the TCID50 value, based on the cytopathic effect caused by this virus in approximately 50% of infected cells. The results, obtained from 4 independent experiments (mean ± SE), are presented as the logTDIC50 values in relation to appropriate DMSO control. * *p* < 0.05 as compared with appropriate DMSO controls.

**Figure 7:**
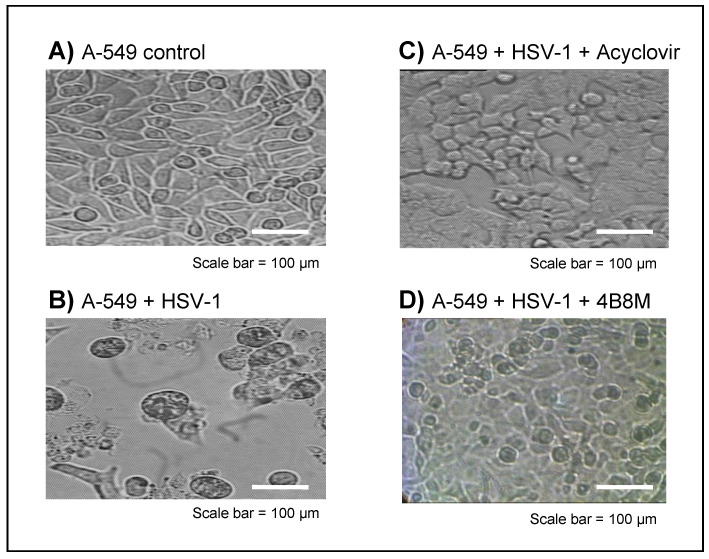
Microscopic evaluation of protective action of 4B8M against HSV-1 and its morphological changes in the A-549 cells. The photographs show 48 h cultures of A-549 cells infected with the HSV-1 virus (magnification 300×). (**A**) Photograph shows a normal appearance of the control culture of A-549 cells in the culture medium; photographs B–D illustrate cells pretreated with the virus: (**B**) HSV-1 negative control after 48 h infection with HSV-1; A-549 cells show some morphological changes, such as loss of monolayer integrity, lyses, granulation, vacuolization in the cytoplasm; (**C**) A-549 cells infected with HSV-1 in the presence of Acyclovir (22 µg/mL); (**D**) A549 cells after 48 h co-incubation with HSV-1 and the peptide (25 µg/mL).

The antiviral effects of 4B8M were also tested in relation to the HSV-1 virus using Acyclovir as a reference drug (Figure 6). An inhibition of HSV-1 replication—virus titers were reduced to 4.6-log—was observed for the peptide at 25 µg/mL and a decrease in the virus titer to 6-log at 12.5 and 6.6-log for 6.0 µg/mL concentration of the compound, respectively—the effects were statistically significant. The efficacy of Acyclovir was stronger and attained its maximal value at 22.5 µg/mL—virus titers were reduced to 1-log. The results shown in the photographs visualize the morphological changes of A-549 cells after infection and the protective effects of 4B8M and the reference drug (Figure 7).

In summary, we demonstrated that the 4B8M peptide exhibits moderate antiviral actions in comparison to the respective reference drugs. It also appeared that the action of the peptide on the replication of the HSV-1 virus was more potent in comparison to its effects on HAdV-5 replication. 

## 3. Discussion

In this investigation, we demonstrated the antiviral properties of the 4B8M peptide in a model of lung epithelial A-549 cells infected with HAdV-5 or HSV-1 viruses using appropriate reference drugs for each virus. The antiviral properties of 4B8M were moderate in comparison with classical drugs, which directly interfere with assembly of viral genetic material [20]. However, taking into account the highest molar concentration of the applied compounds, the concentrations of the reference drugs were somewhat higher, i.e., about 100 µM and 72 µM for Acyclovir and Cidofovir, respectively, in comparison to a 49.7 µM concentration of 4B8M. In addition, in this work, we demonstrated that Acyclovir, at a certain concentration range, inhibited the division of lymphocytes, which suggests some limitations of its use at higher in vivo doses. A clear advantage of a potential use of 4B8M is that, in addition to the inhibition of viral replication, the compound exhibits anti-inflammatory properties [5], thus rendering this compound effective, for example, in the amelioration of viral lung inflammation. Since other potential mechanisms of the peptide’s antiviral action are unknown, we could only ascribe its antiviral property to the ability of the peptide to induce COX-2 together with the down-regulation of EP1/3receptor expression stimulated by lipopolysaccharide [5]. The regulatory actions of PGE2 in viral infection were recently summarized [23]. The inhibitory effects of PGE2 in viral replication were also reported in the infection of A-549 cells by the influenza virus [24,25]. Of interest, viral replication is associated with the induction of autophagy by viruses, and blocking autophagy suppressed viral replication [26]. It appears that autophagy in infected macrophages can also be inhibited by PGE2 via the EP4 receptor [27]. Viral pneumonia infection has consequences, even after the resolution of the disease [28], by rendering mice more susceptible to experimental ovalbumin (OVA)-induced pneumonia [29]. We envisage that 4B8M, apart from the direct antiviral action described in this work, may be protective in cases of lung inflammation from other cases. It was shown that PGE2, derived from lung epithelium, was responsible for maintaining endothelial barrier integrity via the EP4 receptor both in physiologic and inflammatory states [30]. Such a scenario is consistent with the mechanism of 4B8M action, which induces COX-2 production in keratinocytes (epithelial cell type), preserves the expression of EP4, but not EP1/EP3 receptors, and strongly inhibits OVA-induced lung inflammation [5]. Of importance, 4B8M was also effective in this model upon intra gastric administration. The inhibition of EP1 receptor expression may also have significance in combatting skin tumors [31]. In fact, 4B8M also has an ability to inhibit the growth of melanoma cell lines (manuscript submitted for publication [32]). Thus, the mechanism of 4B8M action can be responsible for the mediation of its several therapeutic properties, such as anti-inflammatory, antiviral and antitumor. The application of cyclic peptides, unlike linear ones, has obvious therapeutic advantages due to their resistance to proteolysis and increased cell membrane permeability [33,34]. The 4B8M peptide appeared to be very effective when applied in an ointment for the amelioration of inflammatory skin changes [5]. Thus, its efficacy in the inhibition of pathologic epithelial lesions elicited by *Herpes* would be also expected. Another desirable feature of 4B8M as a potential drug, is its lower toxicity towards mouse splenocytes in comparison to its parent compound CLA. The lack of toxicity against the reference A-549 cell line at 25 μg/mL and towards proliferating splenocytes was also found. In summary, the previously described anti-inflammatory effects of this cyclic tetrapeptide [5] can be extended to antiviral actions, which demonstrates a possibility of a more universal application in preventing inflammation associated with viral infection. 

## 4. Materials and Methods 

### 4.1. Chemistry 

#### 4.1.1. Reagents for 4B8M Peptide Synthesis, Cyclization and Purification

All organic solvents and reagents were of analytical or higher grade and were used as purchased. Acetonitrile for HPLC analysis and purification was HPLC gradient grade. Water for HPLC was purified using MiliQ apparatus. Fmoc-*β^3^*-HoPhe-OH was purchased from Fluka, Darmstadt, Germany; Fmoc-Phe-Wang resin and Fmoc-Pro-OH and all peptide chemistry reagents were purchased from IRIS, Marktredwitz, Germany.

#### 4.1.2. Synthesis of the 4B8M Peptide

**Scheme 1:**
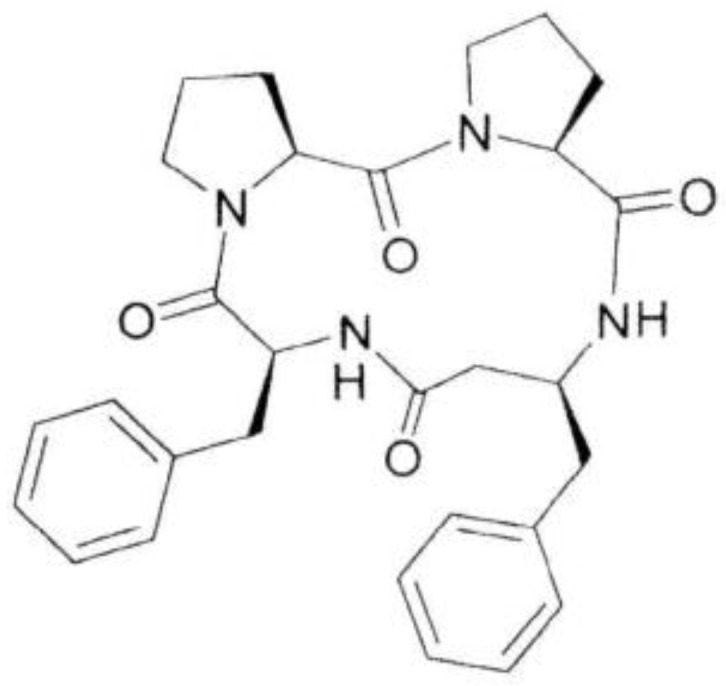
The structural formula of 4B8M (CAS 1332349-13-9, (3S,9S,13S,16S)-9,13-dibenzyl-1,7,10,14-tetraazatricyclo[14.3.0.0^3,7^]-nonadecane-2,8,11,15-tetrone).

The cyclic tetrapeptide (cyclo(Pro-Pro-*β^3^*-HoPhe-Phe)) was synthesized according to the US patent [35] by Janusz Zabrocki et al., Lodz University of Technology, Lodz, Poland. The structural formula of the 4B8M peptide is depicted in Scheme 1. The lyophilized peptide was stored frozen at −20 °C until use; however, we found such conditions unnecessary. In a peptide durability test, after storage for more than 3 years at room temperature in an open vial, the compound showed no traces of decomposition compared to the original sample (HPLC, MS).

#### 4.1.3. Physicochemical Characteristics of 4B8M Peptide

M.W. calculated for C_29_H_34_N_4_O_4_ = 502.61 g/mol, found 503.59 [MH^+^] on a Kratos Kompact Probe MALDI-MS machine (Kratos Analytical Ltd., Manchester, UK). 

Two-dimensional NMR spectra were collected, but will be presented in a separate publication (manuscript in preparation) together with a structure calculated from X-ray data from a single crystal.

Optical rotation [α]_D_ -113.0^o^ (20 °C, c 0.1 in acetic acid 99%), 

HPLC purity >99.5%, UV detection at λ 214 nm, mobile phase A: 0.05% TFA in water (MiliQ purified), B: 0.038% TFA in 82% acetonitrile/water;

t_R_ 12.00 min on Kinetex C_18_ column 150 mm × 4.6 mm, 2.6 µm, 100 Å, gradient 20–60% B in A in 20 min, flow rate 1.25 mL/min, t_R_ 6.56 min on Kromasil C_8_ column 250 mm × 4.6 mm, 5 µm, 100 Å, gradient 50–80% B in A in 15 min, flow rate 1 mL/min, t_R_ 5.93 min on Vydac C_18_ column 250 mm × 4.6 mm, 5 mm, 300 Å, gradient 40–70% B in A in 15 min, flow rate 1 mL/min. 

TLC on Sigma-Aldrich plates, #56524 in MeCN:CHCl_3_:AcOH 8:1:1, R_f_ 0.75; no other spots detected (UV 254) at a concentration of 4 mg/mL.

### 4.2. Biology

#### 4.2.1. Mice

CBA mice were delivered by the Breeding Centre of Laboratory Animals at the Institute of Occupational Medicine, Lodz, Poland, and DBA1 mice were derived from the animal facility of The Institute of Immunology, Wroclaw, Poland. The animals were used only as organ donors, which does not require approval of an ethics committee according to Directive 2010/63/EU of the European Union Parliament and of the Council of 22 September 2010 on the protection of animals used for scientific purposes.

#### 4.2.2. Reagents for Biological Assays

RPMI-1640 medium was purchased from Biowest (Nuaillé, France). Fetal calf serum (FCS) was from HyClone (Pittsburgh, PA, USA). Other reagents, such as L-glutamine, penicillin and streptomycin solution, sodium pyruvate, 2-mecraptoethanol, Cyclolinopeptide (CLA), dimethyl sulfoxide (DMSO) and MTT (3-[4,5-dimethylthiazol-2-yl]-2,5-diphenyltetrazoliumbromide), were purchased from Sigma-Aldrich (St. Louis, MO, USA). Cidofovir (M.W. 279.2) was purchased from Cayman Chemical (Ann Arbor, MI, USA) and Acyclovir (M.W. 225.2) was obtained from Merck (Darmstadt, Germany). Cyclosporin A (CsA) (Sandimmun, Neoral in ampoules) was from Sandoz (Basel, Switzerland).

#### 4.2.3. Preparation of 4B8M Peptide and Reference Compounds for In Vitro Studies

DMSO was used as a solvent for the initial dissolution (5 mg of cyclo(Pro-Pro-*β^3^*-HoPhe-Phe) in 1 mL of DMSO) of the preparation. Then, the peptide was further diluted in the culture medium. Respective dilutions of DMSO in the culture medium, corresponding to appropriate concentrations of the peptide, represented control cultures. For example, for 4B8M concentrations of 25, 12.5 and 6 µg/mL, the DMSO dilution equivalents were 0.5, 0.25 and 0.12%, respectively. DMSO was used also as a solvent for the initial dissolution for Acyclovir; then, the Acyclovir was further diluted in the culture medium. Cidofovir (recommended by supplier for initial dissolution in aqueous media and not soluble in DMSO) was dissolved in PBS. Thus, the effects of 4B8M and Acyclovir were compared with cultures containing respective DMSO dilutions, and those of Cidofovir with the culture without any addition (medium only).

#### 4.2.4. Cell Line and Viruses

A-549: A human lung adenocarcinoma cell line (ATCC CCL185) were from the collection of cell lines from the Institute of Immunology and Experimental Therapy, Wroclaw, Poland. Human *Herpes simplex* virus type-1 (HSV-1), MacIntyre strain; ATTC VR-539™; *Herpesviridae*, DNA enveloped virus; and human adenovirus C serotype 5 (HAdV-5), Adenoid strain 75; ATTC VR-5™; *Adenoviridae* DNA non-enveloped virus were from the Laboratory of Virology, Institute of Immunology and Experimental Therapy, Wroclaw, Poland. The A-549 cell line is routinely used as an efficient, practical and economical cell system for the evaluation of the replication of these virus types [36].

#### 4.2.5. Evaluation of Toxicity of 4B8M Peptide against A-549 Cells

4B8M was initially dissolved in DMSO; further dilutions of the compound were performed in RPMI-1640 medium supplemented with 2% FCS. 4B8M was tested at a 6.0–25.0 μg/mL concentration range. The cytotoxicity of 4B8M was determined by measuring the growth of human lung cancer epithelial A-549 cells (criteria of toxicity effect based on changes in cell morphology (according to: EN ISO10993-5:2009). Biological evaluation. Part 5: Test for in vitro cytotoxicity, International Organization for Standardization, Geneva, Switzerland, 2009). The evaluation of the potential cytotoxic action of 4B8M was performed in a monolayer culture of the A-549 cell line. The cells, at a density of 5 × 10^4^/well, were incubated for 24 h in a cell culture incubator. Following the incubation, the culture supernatants were removed and to the monolayer cell cultures’ appropriate dilutions of 4B8M in the culture medium (200 μL/well) were added and incubated for an additional 72 h. Control cultures contained corresponding dilutions of DMSO. The cell viability was determined by MTT colorimetric assay [37]. The results are presented as the mean optical density (OD) at 550/630 nm ± standard error (SE) from quadruplicate determinations.

#### 4.2.6. Evaluation of Toxicity of 4B8M Peptide against Mouse Splenocytes

The spleens of CBA mice were pressed against a plastic screen into a 0.83% NH_4_Cl solution to lyze erythrocytes (5 min incubation at room temperature). The cells were then washed twice with Hanks’ medium, passed through a glass wool column to remove debris and re-suspended in the culture medium, referred to below as the culture medium, consisting of RPMI-1640, supplemented with 10% FCS, L-glutamine, sodium pyruvate, 2-mecraptoethanol and antibiotics. Splenocytes at a density of 2 × 10^5^/well in 96-flat-bottom culture plates, re-suspended in the culture medium, were cultured for 24 h in a cell culture incubator with the compounds at indicated concentrations. Control cultures contained the solvent (DMSO) at dilutions equivalent to those present in respective doses of the studied compounds. Cell viability was determined by the MTT colorimetric method [37]. The results are presented as the mean OD at 550/630 nm ± SE from quadruplicate determinations.

#### 4.2.7. Evaluation of the Activity of the 4B8M Peptide in the Proliferation Test of Mouse Splenocytes

Mouse splenocytes were prepared as described above. The cells were then distributed into 96-well flat-bottom tissue culture plates at a density of 2 × 10^5^/well. 4B8M and the reference drugs were added at doses indicated in the figure legend. ConA (2.5 µg/mL) was used to induce cell proliferation. Control cultures contained the solvent (DMSO) at dilutions equivalent to those present in respective doses of the studied compounds. Following a 3-day incubation period, cell proliferation was determined using the colorimetric MTT assay [37]. The results are presented as the mean OD at 550/630 nm ± SE from quadruplicate determinations.

#### 4.2.8. Determination of 4B8M Peptide Antiviral Activity

In order to determine antiviral activities, 4B8M was tested using A-549 cells infected with HSV-1 or HAdV-5. Briefly, the A-549 cells were seeded in 96-well culture plates at a density of 1 × 10^5^ cells/mL, and then incubated at 37 °C in a cell culture incubator for 24 h to produce a semi-confluent monolayer. Subsequently, A-549 cells were infected with HSV-1 at a multiplicity of infection (MOI) of 4 or HAdV-5 MOI of 1. Following incubation for 1 h in a cell culture incubator to allow virus adsorption to the cells, the medium (virus inoculum) was removed and the tested compounds or control solvent (DMSO) were added to the infected cells. Acyclovir was used as a reference drug for HSV-1 and Cidofovir for HAdV-5. Non-treated cells and cells treated with HSV-1 or HAdV-5 served as controls. Following 48 h of incubation, the supernatants were collected and stored at −80 °C for virus titration using a standard Tissue Culture Infective Dose 50 (TCID50) method with a two-fold serial dilution. Briefly, the supernatants were diluted and adsorbed to A-549 cells at a density of 1×10^5^ cells/mL in 96-well culture plates. The plates were incubated in 37 °C/CO_2_ atmosphere. Following 48 h of incubation, the viral cytopathic effect (CPE) was analyzed in an inverted microscope. The TCID50 was calculated by determining the end-point dilution of the virus where 50% of the infected cells displayed CPE. The antiviral activity of the tested compounds was determined by comparing the logarithmic reduction factor (log 10) of the viral titer with the DMSO control. The results obtained from 4 independent experiments are presented as logTDIC50 mean values ± SE.

#### 4.2.9. Statistics

All experiments regarding the activities of 4B8M were performed at least 3 times with similar results. The results of a selected experiment are shown in Figure 1, Figure 2 and Figure 3, and presented as the mean values ± SE obtained from quadruplicate determinations (wells). The results obtained from 4 independent experiments are shown in Figure 4 and Figure 6 and presented as the mean values ± SE.

The Brown–Forsythe test was used to determine the homogeneity of variance between groups. When the variance was homogenous, analysis of variance (One-way ANOVA) was applied, followed by post hoc comparisons with the Tukey’s test to estimate the significance of the difference between groups. Nonparametric data were compared using the Kruskal–Wallis test with Mann–Whitney U test to estimate the significance of the difference between groups with the Benjamini–Hochberg correction for performing multiple comparisons. Significance was determined at *p* < 0.05. The statistical analysis was performed using STATISTICA 7.0 for Windows.

## Data Availability

Data are available from authors on request.

## Sample Availability

Samples of the compounds are available from the authors.
